# In vitro modeling of blood–brain barrier and interface functions in neuroimmune communication

**DOI:** 10.1186/s12987-020-00187-3

**Published:** 2020-03-30

**Authors:** Michelle A. Erickson, Miranda L. Wilson, William A. Banks

**Affiliations:** 1 Geriatric Research Education and Clinical Center, VA Puget Sound Healthcare System, Seattle, WA 98108 USA; 2 Division of Gerontology and Geriatric Medicine, Department of Medicine, University of Washington, Seattle, WA 98104 USA

**Keywords:** Blood–brain barrier, Chemokines, Cytokines, In vitro, Leukocyte trafficking, Neuroimmune, Neuroinflammation, Neurovascular unit

## Abstract

Neuroimmune communication contributes to both baseline and adaptive physiological functions, as well as disease states. The vascular blood–brain barrier (BBB) and associated cells of the neurovascular unit (NVU) serve as an important interface for immune communication between the brain and periphery through the blood. Immune functions and interactions of the BBB and NVU in this context can be categorized into at least five neuroimmune axes, which include (1) immune modulation of BBB impermeability, (2) immune regulation of BBB transporters, secretions, and other functions, (3) BBB uptake and transport of immunoactive substances, (4) immune cell trafficking, and (5) BBB secretions of immunoactive substances. These axes may act separately or in concert to mediate various aspects of immune signaling at the BBB. Much of what we understand about immune axes has been from work conducted using in vitro BBB models, and recent advances in BBB and NVU modeling highlight the potential of these newer models for improving our understanding of how the brain and immune system communicate. In this review, we discuss how conventional in vitro models of the BBB have improved our understanding of the 5 neuroimmune axes. We further evaluate the existing literature on neuroimmune functions of novel in vitro BBB models, such as those derived from human induced pluripotent stem cells (iPSCs) and discuss their utility in evaluating aspects of neuroimmune communication.

## Background

The vascular blood–brain barrier (BBB) is primarily comprised of highly specialized endothelial cells that regulate communication between the brain and immune systems. The BBB mediates neuroimmune communication in many complex ways which can be categorized into 5 axes as described in a recent review [1]. The 5 axes are: (1) modulation of BBB impermeability, (2) immune regulation of BBB transporters, secretions, and other functions, (3) transport, penetration, and uptake of neuroimmune-related substances, (4) immune cell trafficking between blood and brain, and (5) BBB secretions of immunoactive substances. In vitro BBB models offer great utility in the study of neuroimmune communication, because they provide a simplified biological system which can be used to study molecular mechanisms that are difficult or not possible to study in vivo. Further, in vitro models of the BBB can be constructed using human tissues [2–4], and thus have translational utility. Studies using conventional in vitro BBB models have already contributed substantially to our understanding of BBB neuroimmune functions, and we dedicate much of this review to summarizing these studies within our conceptual framework of the 5 neuroimmune axes.

In the past decade, in vitro modeling of the BBB has advanced due to the development of new models that use brain endothelial-like cells derived from human induced pluripotent stem cells (iPSCs) [4], novel 3D culture systems that grow on a tubular matrix and can be studied under flow conditions [5], vascularized brain organoids [6, 7], and co-culture systems that incorporate multiple cell types of the neurovascular unit [4]. In the last section of our review, we will evaluate recent advances of in vitro BBB models and their utility in studying neuroimmune axes.

### Properties of the BBB that confer its barrier and interface functions

**Fig. 1:**
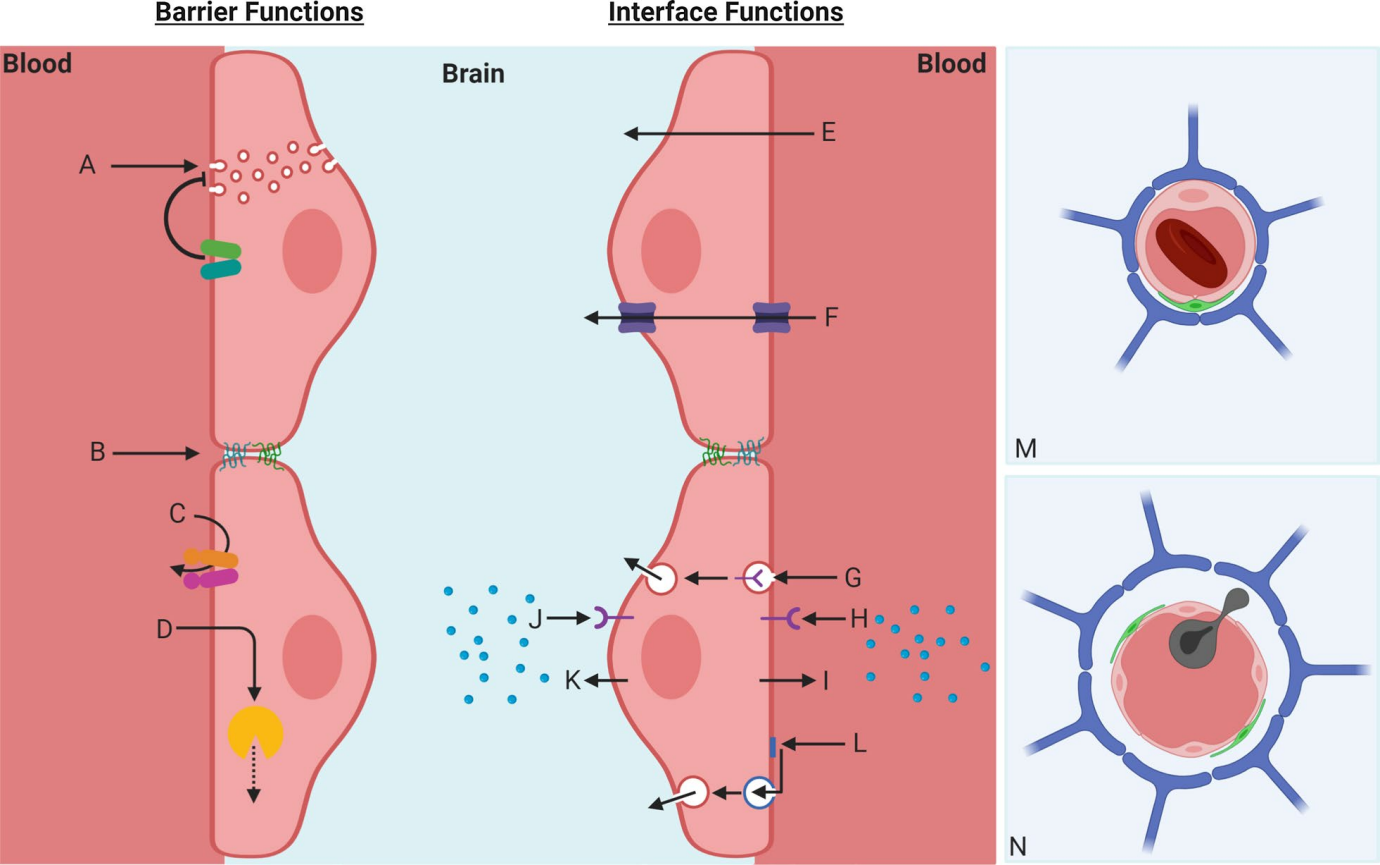
Barrier and interface functions of the vascular BBB. Left panel—barrier functions of brain parenchymal capillaries are shown on the left and include **a** suppression of micropinocytosis via lipid transporters such as Msfd2a which prevents transcellular leakage, **b** expression of tight junction proteins that prevent paracellular leakage, **c** expression of efflux transporters which prevent the diffusion of hydrophobic substances and certain xenobiotics across the BBB, and **d** expression of metabolic enzymes which degrade substances taken up by the endothelium. Interface functions are shown on the right and include **e** transcellular diffusion of substances that are membrane permeant, **f** facilitated diffusion or active transport through solute carriers, **g** transport via receptor-mediated transcytosis, secreting (**i**, **k**) or responding to secretions (**h**, **j**) in the blood or brain compartments, and **l** inducing uptake and transport via adsorptive transcytosis. Right upper panel **m** depicts the positioning of the brain capillary (pink) with an associated pericyte (green) and astrocyte endfeet (blue). Note the relative absence of perivascular space, and red blood cell in the capillary lumen. Right lower panel **n** illustrates a post-capillary venule which is the predominant site of leukocyte trafficking across the vascular BBB. Diapedesis of a leukocyte (grey) into the perivascular space is depicted. This figure was created with BioRender

Barrier functions of the BBB are conferred by specialized features of brain endothelial cells (BECs), which prevent the unregulated passage of blood-borne substances into the brain. These features include (1) the expression of tight junction proteins (TJPs) including claudins, occludins, and junctional adhesion molecules (JAMs) which prevent the paracellular diffusion of substances [8], (2) suppression of pinocytic vesicles and fenestrae, which prevents transcellular pathways of diffusion [9, 10], (3) expression of efflux transporters, which actively inhibit the passage of substances that would otherwise diffuse across brain endothelial cell membranes [11], and (4) expression of enzymes that metabolize bioactive substances before they can reach the brain [1, 12]. The BBB also serves as an interface that regulates the transport of molecules between blood and brain compartments. Transport of molecules across the BBB occurs through different mechanisms that include passive diffusion across membranes, passive or active transport via solute carriers, active transport via receptor-mediated transcytosis, and induction of transport via membrane interactions, including adsorptive transcytosis [1]. Finally, the BBB acts as a signaling interface in that it can respond to signals that arise from the blood or brain compartment, and transmit signals through secretions into either compartment [12], or through other mechanisms such as matrix interactions [13]. These properties of the BBB are illustrated and described further in Fig. 1.

Brain endothelial cells are located in close apposition to other brain cell types that are important in regulating their functions, including astrocytes, pericytes, neurons, microglia/perivascular macrophages, oligodendrocytes, and mast cells [1]. Acellular components such as extracellular matrix and glycocalyx also contribute to barrier and interface functions of brain endothelial cells [1]. Collectively, these cellular and acellular components comprise the neurovascular unit (NVU). We refer readers to recent, comprehensive reviews of the NVU with reference to its contributions to overall BBB function, and neuroimmune functions for in-depth evaluation [1, 14, 15]. In vitro models of the BBB often incorporate components of the NVU in co-culture, most commonly astrocytes and/or pericytes, which will be discussed below in more detail.

Astrocytes are the most abundant cell type in the brain and regulate multiple physiologic processes including synaptic and neuronal functions, functional hyperemia, convective flow of brain interstitial fluid, and induction and maintenance of the brain endothelial phenotype [16–18]. Astrocyte end feet surround brain capillaries, arterioles, and venules and coverage of these vessels was shown to be almost 100% [19]. In vitro, astrocytes strengthen BEC barrier properties and enhance expression of transporters, such as P-glycoprotein (P-gp) and GLUT1, as well as metabolic enzymes [17]. In vitro studies have also demonstrated that culture conditions that allow direct contact of astrocytic processes and the endothelial monolayer facilitate a stronger barrier, although secreted factors from astrocytes also contribute to the BBB phenotype [20], including immune responses [18]. In summary, astrocyte co-culture with BECs in vitro can promote both barrier and interface functions of BECs.

Pericytes also contribute to development and maintenance of the vascular BBB. They share a basement membrane and make direct contact with BECs via peg and socket as well as gap junctions, and are the most closely apposed to BECs vs. other NVU cell types [21]. During embryonic development, pericyte attachment to BECs induces BBB tightening by downregulating genes that are associated with pinocytic vesicle formation [9, 22]. Pericytes are also important for the induction of the BBB phenotype in vitro, as pericyte coculture with BECs increases the integrity of the barrier and efflux transporter functions [23, 24].

Although the specific neuroimmune functions of pericytes, astrocytes, and other non-endothelial cells of the NVU are not the main focus of this review, they are clearly important contributors to neuroimmune functions of the BBB [1]. We will periodically refer to studies in later sections which consider pericyte and astrocyte interactions with the brain endothelial cells in vitro.

### Conventional in vitro models of the BBB

**Fig. 2:**
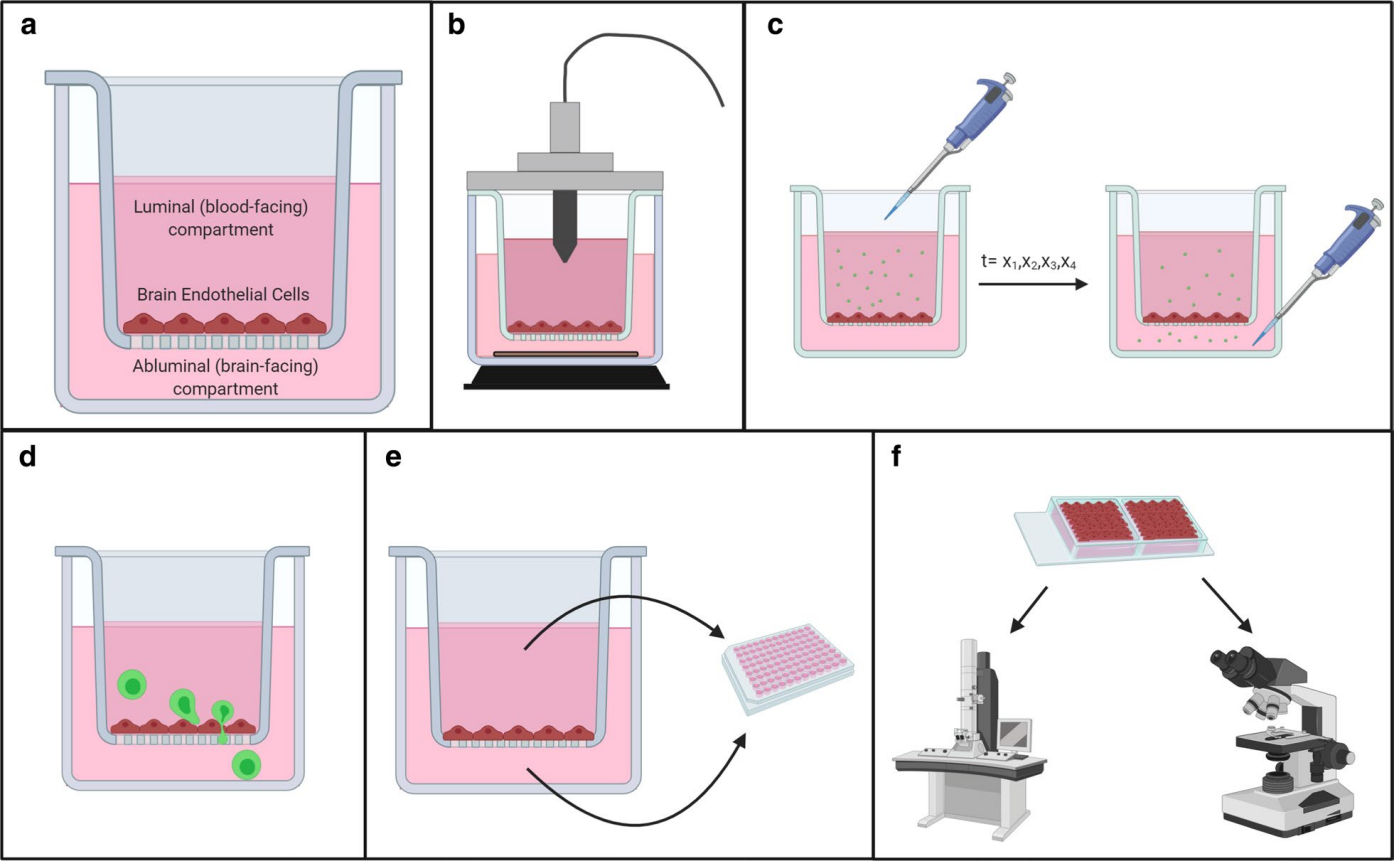
Evaluating neuroimmune axes using conventional 2D cultures of brain endothelial cells. **a** A conventional 2D model of brain endothelial cells in monoculture grown on a transwell. **b** Measurement of TEER using an Endohm cup chamber apparatus to evaluate BBB integrity and disruption (Axis 1), **c** Measurement of Pe. A fluorescent tracer is pipetted into the luminal chamber and medium is collected from the abluminal chamber over a time interval and evaluated for fluorescence. This method can be used to evaluate BBB disruption when inert tracers are used (Axis 1), and transport of labeled molecules and/or vesicles (Axis 2 and 3). **d** Measurement of immune cell interactions and trafficking. Leukocytes can be labeled and added to the upper chamber for evaluation of their interactions with brain endothelial cells (Axis 4). **e** Polarized secretions from brain endothelial cells can be measured using standard assays like ELISAs at baseline (Axis 5) and following an immune stimulus (Axis 2). **f** Brain endothelial cells grown on appropriate materials for imaging can be fixed and/or stained for expression/localization of tight junction proteins or vesicular changes and imaged by standard light/fluorescence microscopy or electron microscopy (Axis 1). This figure was created with BioRender

Conventional in vitro modeling of the BBB is generally conducted by isolating and culturing primary BECs or using immortalized BEC lines grown in 2D as a flat monolayer. For studies evaluating BBB leakage, transport, or polarized secretions, cells are most often cultured on a transwell insert which allows for the evaluation of transendothelial electrical resistance (TEER) of the monolayer, the permeability of tracers across the monolayer, and sampling of the luminal (blood-facing) and abluminal (brain-facing) compartments (Fig. 2). The choice of brain endothelial cell type (primary vs. immortalized and from which species) to be used for in vitro modeling is important, because each offers different advantages and limitations [25]. For example, primary brain endothelial cells cultured in vitro can replicate important aspects of the BBB phenotype such as high TEER and low permeability to inert tracers [25], but yield tends to be low when these cells are isolated from commonly used smaller lab animals such as rodents. BECs isolated from larger animals such as cows or pigs provide higher yields due to their bigger brains, but can be difficult to obtain and to study due to a lack of resources to use large animals and limited availability of species-compatible reagents such as antibodies and recombinant proteins. Primary BECs from humans are difficult to obtain in sufficient quality and quantity, and usually require isolation from fresh, surgically resected tissues from living patients to reliably develop high TEER [2]. Most of these BECs are from donors receiving temporal lobe resective surgery for intractable epilepsy, but advances in surgical techniques such as gamma knife radiosurgery may diminish availability of tissue for primary BEC isolation [26]. Although many BBB properties have been extensively evaluated in surgically-derived human BECs [27, 28], there is some question whether BECs derived from healthy brain tissues of epileptic patients would perform similarly to those of healthy humans [29], and this issue may also vary by donor. Primary human brain microvascular endothelial cells are also available commercially, but these are costly, of limited availability, and are usually derived from fetal tissues [5]. Their ability to form a tight BBB can be variable and often not well-characterized by the suppliers. Primary BECs from non-human primates such as rhesus macaques have also been developed and used to study immune responses of brain endothelial cells [30]. Immortalized brain endothelial cell-derived cell lines have been developed from mouse, rat, and human tissues [3, 31, 32], and offer the benefit of their ease of use, expression of tight junction proteins and other BBB-specific proteins, and high yield for experiments that require more starting material. However, these cell lines generally develop TEER < 100 Ω*cm^2^ [3, 25], which is not sufficiently high to study BBB transport without a substantial influence of BBB leakage [33] and may also be problematic in studying polarized secretions since secreted factors could leak from one side of the monolayer to the other.

## Use of conventional in vitro BBB models to study the neuroimmune axes

In vitro models are useful for studying neuroimmune communication because they can recapitulate many immune functions of BECs that are known to occur in vivo. Below, we discuss how conventional in vitro BBB models have been used to understand the neuroimmune axes. Although we describe each axis separately, we emphasize that this is done to provide a conceptual framework, and that more than one axis could be operational in a given immune process. Some examples of how the axes may be thought of as integrated are also highlighted in this section.

### Axis 1—Modulation of BBB impermeability

The relative impermeability to molecules that do not freely diffuse across membranes is perhaps the most widely recognized feature of the BBB, and is essential for maintaining brain homeostasis. Disruption of the BBB, which we define here as loss of impermeability to molecules due to leakage, can be induced by a number of immune factors including cytokines and chemokines [15, 34], microbes and their components [35–37], complement proteins [38], acute phase proteins [39], etc. A few methods are available to evaluate BBB disruption in vitro, which we discuss in-depth in this section. Further, we provide some examples in the literature on use of in vitro models to evaluate BBB disruption in response to immune factors.

#### Mechanisms and measurement of BBB disruption in vitro

Just as BBB impermeability depends on acquisition of intercellular tight junctions, loss of fenestrae, and decreased pinocytosis, so in theory, BBB disruption can occur by paracellular and transcellular/transcytotic pathways. BBB disruption is generally considered to be a pathological process that results in increased paracellular and/or transcellular diffusion of substances due to reduced functions of TJPs and/or increased vesicular mechanisms, respectively [1]. However, some workers in the field feel that there may be a physiological variation in tightness of the barrier. It has been shown recently that there are at least regional variations in BBB features among brain microvascular segments, with small brain arterioles having an abundance of caveolar vesicles that are virtually absent in brain capillaries [40]. These arteriolar vesicles were shown to mediate neurovascular coupling, and thus have physiological functions. In some instances of more severe injuries, BBB disruption also occurs due to degeneration and loss of brain endothelial cells, resulting in cerebral microbleeds [1].

In conventional 2D culture systems where endothelial cells are grown on transwells, disruption can be measured by evaluating TEER, as well as the permeability of inert tracers across the monolayer in the blood-to-brain direction. TEER evaluates the electrical resistance of the monolayer, which requires a patent channel between the luminal and abluminal transwell chambers. It is mainly influenced by tight junction protein integrity and can be measured using a voltohmmeter, or through impedance spectroscopy [41]. Voltohmeters that are typically used to measure TEER (e.g. the EVOM2) apply an alternating current of ± 10 μA at a single frequency of 12.5 Hz. Specialized electrodes are used that simultaneously apply current and measure voltage and come in two formats: the STX2 or “chopstick” electrode pair and the EndOhm meters. The STX2 is manually positioned with one electrode in the upper and one electrode in the lower chamber for TEER measurement. TEER readings with STX2 electrodes depend on the electrode positions, and the uniformity of the current density can have a substantial effect on TEER measurements—overestimation of TEER may occur as a result of uneven current density when larger transwell inserts are used [41]. The STX electrodes must also be carefully handled to ensure consistent placement, and no cell damage during measurement. EndOhm chambers serve as an alternative to STX electrodes—these chambers have electrodes with fixed geometries, and the transwell is placed into the chamber for TEER measurement, resulting in more uniform current density across the membrane. As a result, there is lower variation in measurement of a given sample in the EndOhm chamber when compared with STX2 electrodes [41]. In general, measurement of TEER using voltohmmeter requires performing measurements outside of the cell incubator, and so fluctuations in medium temperature may contribute to variability of the measurements if the plates are not equilibrated. Impedance measurement systems use a different approach for measuring TEER, and can also evaluate properties like cell membrane capacitance which is an indicator of cell attachment and growth. In these systems, AC voltage is applied with a frequency sweep and the amplitude and phase of the resulting AC current is measured. Electrical impedance is then calculated as the ratio of the voltage–time function and the current–time function [41, 42]. Like resistance, impedance is expressed in ohms and can be normalized to the growth surface area. An additional benefit of commercially available impedance measurement systems is that they offer real-time measurements without the need to remove the cells from the incubator.

TEER is conventionally expressed as the resistance in ohms (Ω) multiplied by the area of the growth surface in cm^2^, and the TEER of a cell-free transwell in cell culture medium is subtracted to calculate the TEER of the cell monolayer [41]. In vivo, the BBB has been estimated to have very high TEER of around 8000 Ω*cm^2^ based on studies that evaluated ion flux from blood to brain in situ [43]. Conventional models of the BBB typically do not achieve TEER values this high, however it has been found using bovine brain endothelial cells that when TEER exceeds about 131 Ω*cm^2^, subsequent increases in TEER result in such minor decreases in permeability of small inert tracers such as sodium fluorescein (376.3 Daltons) that the relation becomes flat (at a permeability threshold of about 0.5 × 10^−6^ cm/s; more details on permeability measurements provided below) [33]. A more recent study using porcine brain cerebral endothelial cells found an inflection point where sucrose (342.3 Daltons) permeability stabilized at a similar level of about 0.5 × 10^−6^ cm/s, but at a higher TEER threshold of about 600 Ω*cm^2^ [44]. In iPSC-derived brain endothelial cells, a higher TEER threshold of around 500 Ω*cm^2^ is needed before the TEER-permeability relation flattens for sodium fluorescein, with the permeability measure stabilizing around 0.5 × 10^−6^ cm/s [45]. The reason for these differences in models is presently unclear, but highlights that similarly designed studies may need to be done as quality controls to evaluate model-specific thresholds of permeability and TEER that indicate sufficient barrier properties for the studies being performed. Conventional 2D BBB models using isolated primary rodent brain endothelial cells can typically achieve TEERs of 100–300 Ω*cm^2^, but TEER values of up to 800 Ω*cm^2^ have been reported under optimized conditions in monoculture (e.g. in the presence of hydrocortisone) and when co-cultured with other cell types of the neurovascular unit such as astrocytes or pericytes or their conditioned medium [25, 46]. Primary cultures of brain endothelial cells from larger animals such as cows or pigs typically have TEERs of around 400–1000 Ω*cm^2^ or higher [25], but apparent species differences may reflect technical factors such as BEC yield or isolation and culture conditions rather than differences in physical tightness of the barrier in vivo.

Although a low TEER indicates a connection between the luminal and abluminal chambers, it does not provide information on the diameter of that connection [25]. Assessment of size-selective BBB leakage in vitro can be done by measuring permeability of inert, hydrophilic tracers that are fluorescent or radiolabeled. Generally, this is done by applying tracers of varying molecular weights to the luminal chamber, and then measuring the amount of tracers that reach the abluminal side over time. The flux of substances across in vitro BBB monolayers is conventionally expressed as the permeability-surface area coefficient of the endothelial monolayer (abbreviated as Pe), expressed in units of cm/min or cm/s [47]. Occasionally, other abbreviations such as Pc and Papp are used to express permeability [44, 48]. Although TEER and Pe tend to be correlated up to a threshold, as described above, TEER is heavily influenced by the path of lowest resistance in the monolayer, whereas Pe depends on the sum of the transport across all pathways. Pe of inert tracers varies by size, with a large tracer like albumin (66.5 kDa) showing about a 30-fold lower Pe value than that of sucrose (340 Da) [35]. Typical Pe values for sucrose in conventional in vitro BBB models is around 0.5–10 × 10^−6^ cm/s in primary cells where TEER ranges from about 300–1000, and tends to be an order of magnitude higher or more in commonly used immortalized cell lines [25].

BBB disruption may follow different modalities, and the abilities of TEER and Pe to detect these modalities should be considered. We note that the discussion that follows is theoretical, and based on principles of Pe and TEER measurements described above rather than on formally published studies. We first posit that TEER does not readily measure BBB disruption that is caused by transcytotic mechanisms such as pinocytosis since these mechanisms do not typically result in a patent channel. As the vesicles are in the 80–100 nm range [40], transcytosis does not display the size-dependent leakage discussed above with Pe, although larger sized molecular tracers can more sensitively detect BBB disruption across transcytotic pathways vs. smaller tracers because they are less permeant at baseline. Conversely, smaller tracers can more readily penetrate paracellular openings and thus tend to correlate with TEER. The degree to which a pathological event results in BBB disruption that is due to paracellular, transcellular, or transcytotic processes can at best be inferred from comparison of measures by TEER and permeability of molecular weight markers. Verification of the predominating disruptive pathways requires their microscopic evaluation. Immunofluorescent imaging of TJPs, for example, can provide insight on their expression and proper localization at cell–cell contacts. However, TJP changes do not always correlate with measures of BBB disruption [49], and so a more comprehensive method of evaluating structural change in BECs, such as electron microscopy, may be required for concluding which mode of disruption predominates.

#### Effects of immune factors on BBB disruption in conventional in vitro BBB models

BECs express cytokine and chemokine receptors, as well as receptors such as toll-like receptor 4 (TLR4) for pathogen-associated molecular pattern molecules like bacterial lipopolysaccharide (LPS), permitting them to respond to these factors [1]. Although there are numerous studies in vivo that have provided insight on how inflammatory molecules contribute to BBB disruption, we limit consideration here to processes that have been specifically explored using in vitro BBB models. In primary BECs, LPS can cause BBB disruption that is associated with TJP dysfunction [35, 50–52] and may cause BEC apoptosis at a higher dose [53]. The effects of LPS on BBB disruption are mitigated by indomethacin, indicating that cyclooxygenases contribute to LPS-induced BBB disruption [35]. Conflicting results have been reported regarding how supportive cells of the NVU influence BEC responses to LPS. Whereas bovine BECs were protected from LPS-induced BBB disruption during co-culture with astrocytes or mixed glia from rats [50], mouse BECs were not protected from LPS-induced BBB disruption when in co-culture with mouse astrocytes, pericytes, or both [35]. Other microbial factors such as HIV-1 and its proteins Tat and gp120 can also contribute to BBB disruption, via mechanisms that involve oxidative stress and modifications of TJPs [36, 54–56] and sensitization to other microbial factors like LPS [57]. Pro-inflammatory cytokines such as tumor necrosis factor-alpha (TNF-α), interleukin-1 beta (IL-1β), and interleukin-6 (IL-6) have been shown to lower TEER [58]. In primary rat BEC cultures in astrocyte conditioned medium, TNF-α and IL-1β were shown to induce transient reductions in TEER by 60 min that recovered after about 210 min. Recovery was attributed, in part, to degradation of the applied cytokines. In contrast, application of IL-6 caused TEER reductions that persisted for at least 300 min. The TEER-reducing effects of all three cytokines were cyclooxygenase-dependent [58]. In bovine BECs co-cultured with astrocytes, increases in permeability to sucrose and inulin induced by TNF-α treatment were delayed, with no increases in permeability being evident until 16-h post-treatment [59]. Primary human microvascular BECs also showed peak reductions in TEER following application of TNF-α, interferon-gamma (IFN-γ), or IL-1β after about 18 h post-treatment [52]. Studies on mechanisms contributing to BBB disruption in inflammatory contexts have largely focused on dysregulation of TJPs, but recent works have also evaluated participation of cellular prion protein [60] and micro-RNA [61]. Chemokines like C–C motif chemokine ligand (CCL) 2 can also mediate BBB disruption under physiological and pathological conditions, such as oxygen–glucose deprivation [34, 62]. It was shown that CCL2 causes subcellular redistribution of claudin-5, occludin, ZO-1, and ZO-2 via serine phosphorylation through a RhoA/protein kinase C-α pathway [63], and also induces claudin-5 and occludin internalization via caveolae-dependent endocytosis [64]. Other inflammatory mediators such as bradykinin, histamine, serotonin, arachidonic acid, and ATP can also increase the paracellular permeability of the BBB [65].

An interesting speculation is whether the BBB and BECs respond to baseline levels of LPS in the circulation. It is clear that under certain non-pathologic conditions such as a high fat meal or jogging that LPS can leak from the gut and contribute to increased LPS concentrations in the blood [66, 67], and there is some speculation that low levels of LPS may circulate physiologically. If so, LPS and the immune system may play roles in the physiologic regulation of the BBB.

In summary, in vitro models of the BBB have provided a useful tool to better understand direct interactions of immune factors such as cytokines and chemokines, LPS, and others that cause BBB disruption. We have highlighted some limitations and technical considerations associated with use of in vitro models to study BBB disruption. BECs in vitro have provided much information on paracellular mechanisms of BBB disruption that are attributed to the modification of TJPs, but much less is known regarding use of in vitro BBB models to evaluate vesicular/transcellular mechanisms of disruption. Transcellular BBB disruption does occur in vivo following inflammatory insults [68, 69], and thus may be an important future avenue of investigation.

### Axis 2—Immune regulation of BBB transporters, secretions, and other functions

In addition to causing disruptive changes at the BBB, immune substances can also alter other functional components of the BBB. Examples include LPS causing altered transporter function or related BEC activities like adsorptive endocytosis and transcytosis [70], LPS or IL-1 inducing BEC secretion of immune factors like PGE2 which are important for the induction of fever [71, 72], and expression of MHCII molecules induced by IFN-γ [73]. Transporters at the BBB can mediate the passage of substances in the blood-to-brain direction (influx), or brain-to-blood direction (efflux), and both influx and efflux transporter activities have been studied using in vitro BBB models [74, 75]. The calculation of Pe that is used to evaluate leakage can also be applied to assess transport of substances across the BBB. Insulin can cross the BBB in the blood-to-brain direction via a saturable influx transporter that is distinct from its receptors [76, 77], and BBB transport is thought to be a crucial component of insulin’s CNS regulatory actions since it is minimally expressed in the brain [78]. The insulin transporter is functional in vitro [74], and has been evaluated for functional changes following LPS treatment of BECs. LPS treatment alone had no significant effect on insulin transport in vitro by BECs, but LPS did increase saturable insulin transport in the presence of lymphocytes. This work showed that LPS affected BBB insulin transport indirectly by inducing nitric oxide transport from other cells types. [74]. The efflux transporter P-gp is also regulated by immune stimuli such as LPS and TNF-α, but in this case they act directly on the BEC. However, the directionality of regulation depends on the stimulus and the model. For example, TNF-α upregulates P-gp expression and function in RBE4 cells, which are immortalized rat BECs [79], whereas LPS functionally downregulates P-gp in primary rat BECs co-cultured with astrocytes [51]. Isolated rat brain microvessels rapidly downregulate P-gp function in response to LPS or TNF-α [80], and LPS was shown to cause P-gp dysfunction in BECs when co-cultured with microglia [81]. Together, these data suggest that the association of glia could negatively affect P-gp function in response to immune stimuli. LPS has also been shown to reduce the expression of the lactate receptor GPR81 and monocarboxylate transporter MCT1 in primary rat BECs, which could have important consequences on BEC metabolism [82]. Finally, LPS can increase the transport of HIV-1 across the in vitro BBB by a mechanism that depends on IL-6 and granulocyte–macrophage colony stimulating factor (GM-CSF) production. Responses to IL-6 and GM-CSF in this same study were polarized, as these cytokines did not enhance HIV-1 transport when applied to the abluminal side of the transwells [83]. Pericyte co-culture further increases HIV-1 transport without influencing TEER through mechanisms that require cross-talk via secretory signals from BECs and pericyte responses to those signals that enhance the BEC permeability to HIV-1 [84]. This process involves a second neuroimmune axis (Axis 5), which will be discussed later in the review.

The above functions of BECs in Axis 2 highlight that immune factors can modulate a variety of BBB functions other than disruption. However, assays that evaluate BBB transporters in vitro must be carefully planned- initial studies must be carried out to confirm that transporter function is present under physiological conditions, and mirrors features of in vivo transport such as saturability, etc. It must also be confirmed that transport can be distinguished from leakage, which can be done by co-evaluating Pe of a similarly sized inert tracer. The influence of leakage would also presumably be mitigated if BECs remain above TEER and below Pe thresholds that are defined for the model (see Axis 1) following treatment. Another limitation to consider is that BECs in culture can lose some of their BBB-specific properties, including expression of transporters, cytokine receptors, and chemokines [85].

### Axis 3—Transport, penetration, and uptake of immune substances

In vivo, the BBB transports a variety of cytokines and chemokines including interleukins (IL)-1α and β, 6, 15, TNF-α, CCL2 and 11, cytokine-induced neutrophil chemoattractant-1 (CINC-1), leukemia inhibitory factor-1 (LIF-1), epidermal growth factor (EGF), and fibroblast growth factor FGFs [70]. However, there is a paucity of literature to indicate whether cytokine/chemokine transport occurs in cultured BECs. In a study that distinguished the cell origins of cytokine secretions by culturing human pericytes and astrocytes with mouse BECs using species-specific cytokine multiplex ELISAs, it was found that pericyte and astrocyte-derived CCL2, GM-CSF, and TNF-α were present at high levels in the luminal compartment [86]. This finding suggests that there is an efflux system for these cytokines. One example of a BBB efflux system that is active in vitro is the Duffy antigen/receptor for chemokines (DARC), also referred to as atypical chemokine receptor 1″ (ACKR1), which can mediate the transport of chemokines such as CCL2 and CCL5 in the brain-to-blood direction [87]. DARC expression may therefore be important in establishing chemokine gradients to facilitate immune cell trafficking across the vascular BBB, and thus also participates in Axis 4 (described below). Further, DARC is upregulated by TNF-α [87], and so is also a component of neuroimmune Axis 2.

Emerging research has shown that extracellular vesicles (EVs) such as exosomes and microparticles can be important in conveying immune signals [88]. EVs can also cross the BBB [89]. For example, exosomes derived from macrophages can be taken up by hCMEC/D3 cells by an energy-dependent endocytic process [90]. Cell adhesion molecules that participate in immune cell trafficking such as intercellular adhesion molecule-1 (ICAM-1) and lymphocyte function-associated antigen-1 (LFA-1) contribute to exosome uptake, as do C-type lectin receptors. Further, macrophage exosome uptake by hCMEC/D3 cells is potentiated by LPS through increased ICAM-1 expression [90], and thus incorporates Axis 2 as another regulatory arm. HEK293-T cell-derived exosomes are also taken up by immortalized BECs via endocytic processes, and their transport across BECs is potentiated by luminal application of TNF-α [91]. Microvesicles from immune cells can also be taken up by BECs, and alter transcriptomic profiles of TJPs, proteolytic processes, and vesicular transport [92].

The cellular mechanisms of transport and uptake of immune substances by endothelial cells is perhaps less well studied in vitro vs. other axes. This is, in part, because the mechanisms that regulate BBB transport and uptake are often poorly understood and so are difficult to therapeutically target. For example, many substances that are transported across the BBB do not use their signaling receptors for transport. When the identity of the transporter is not known, selective inhibition strategies are difficult to design. The benefit of in vitro models is that they might offer a way to identify novel transporters of immune factors, assuming that they are functional in the model. BECs in vitro also offer the ability to study specific mechanisms of uptake, since manipulating endocytic processes is much easier to do in vitro vs. in vivo.

### Axis 4—Immune cell trafficking between blood and brain

Immune cell trafficking across the vascular BBB is thought to be minimal under physiological conditions [93, 94], although immune surveillance of the CSF does occur [1]. Under pathological conditions such as HIV infection, multiple sclerosis, stroke, and traumatic brain injury, the vascular BBB is an important interface for immune cell trafficking. In vitro BBB models have been used to study trafficking of neutrophils, macrophages, and T-cells across BECs [95], and have provided molecular insight into how these processes occur. Adhesion molecules that modulate immune cell trafficking in vivo such as ICAM-1, ICAM-2, and VCAM-1 are expressed by mouse BECs, and when BECs are co-cultured with co-mixed glia, and ICAM-1 and vascular cell adhesion protein-1 (VCAM-1) are upregulated following LPS treatment [96]. Selectins, which mediate the initial capture and rolling of leukocytes, are also expressed by BECs in vitro [97]. Diapedesis, which is the complete passage of the leukocyte across the BBB, may occur as a result of the immune cell traveling between cell–cell junctions (paracellular) or by tunneling through specific membrane structures in the cell (transcellular). There is evidence for both pathways [95], and in vitro models have elucidated mechanisms that regulate leukocyte trafficking via either route. Regarding leukocyte trafficking and other BBB properties, it has been found that inducing shear stress, which is normally encountered as a result of blood flow across brain endothelial cells in vivo, can cause some important changes in BEC phenotype. For example, sheer stress induced higher TEER, mRNA upregulation of TJPs and BBB transporters, as well as increased expression of mRNA levels of cell adhesion molecules such as VCAM-1, ICAM-1, and PECAM [98]. In contrast, P- and E-selectin mRNAs were decreased, which may further indicate a BBB phenotype since BECs lack P- and E-selectins in their Weibel-Palade bodies at baseline [99]. Treatments of primary mouse BECs with TNF-α or IL-1β under flow conditions that induced sheer stress induced increases in both ICAM-1 and VCAM-1. IL-1β was more potent than TNF-α on inducing either adhesion molecule. Further, IL-1β treatment showed a dose-responsiveness for ICAM-1 upregulation between 0.05 and 0.5 ng/ml, whereas VCAM-1 increases were equal in magnitude across all doses. Higher induction of ICAM-1 and VCAM-1 was associated with less T-cell crawling and more diapedesis. Higher levels of ICAM-1 and VCAM-1 upregulation were associated with greater reductions in TEER, but higher levels of transcellular diapedesis. This finding was contrary to the assumption that reduced TEER would be associated with greater paracellular diapedesis due to permissiveness of TJPs, and instead, indicated that high cell surface levels of ICAM-1 promote transcellular diapedesis. VCAM-1 expression was found not to promote either pathway [100].

Primary and immortalized (bEnd.5) mouse BECs have been compared in terms of their suitability for studying molecular mechanisms of T-cell trafficking [101]. In this study, bEnd.5 cells were more leaky and failed to show proper localization of occludin to cell junctions. Surprisingly, neither primary mouse BECs nor bEnd.5 cells showed increased permeability in response to TNF-α treatment, but did upregulate their expression of VCAM-1 and ICAM-1 following TNF-α treatment. bEnd.3 cells have also been used to illustrate the importance of flow in immune cell trafficking: in 2D-culture without flow, T-cell adhesion to both BEC types was similar under basal and TNF-α stimulated conditions. T-cell arrest following TNF-α treatment under flow-conditions was also similar in primary mouse BECs vs. bEnd.5 cells. In contrast, diapedesis at baseline and under TNF-α stimulated conditions was much higher in bEnd.5 cells vs. primary mouse BECs under static culture, and under flow conditions. The reduction of diapedesis in primary cells vs. bEnd.5 was associated with an increased crawling distance and crawling velocity, which was attributed to a lower frequency of sites permissive for diapedesis on primary cells [101]. In vitro models have also been used to evaluate monocyte migration across the BBB in context of a number of pathophysiological conditions/pharmacological manipulations such as HIV-1 infection, dopamine elevations, and cannabinoid 2 receptor activation [102–104].

The regulation of immune cell trafficking across the BBB is an important, yet complex process that is highly unique in BECs vs. vasculature in the periphery. In vitro BEC models have provided insight on mechanisms of adhesion molecule induction, the relative importance of different adhesion molecules on leukocyte trafficking, relative differences in cell lines, and the importance of sheer stress on aspects of leukocyte trafficking. One area of development in Axis 4 is the evaluation of immune cell trafficking across other brain interfaces, such as across choroid plexus epithelial cells. In vitro models have evaluated primary vs. cell lines of choroid plexus epithelial cells [105] and found that the trafficking of human T-cell subsets differed in human BECs vs. a choroid plexus epithelial cell line under baseline and inflammatory conditions [106]. Given the importance of these barriers in immune surveillance, responses to injury, and other CNS functions [1], development of novel in vitro models to study them could be an important approach to advance the field.

### Axis 5—Immune secretions of barrier cells

BECs and other cells of the NVU not only respond to immune stimuli, but also secrete immune factors which are important in mediating autocrine functions as well as cross-talk among cells of the NVU, and may also mediate communication between the brain and blood if a signal from one side results in a secretion from the other. In vitro BBB models have been especially useful in identifying factors secreted by BECs, other NVU cells types, and the downstream activities mediated by secreted immune factors. The cytokines granulocyte colony stimulating factor (G-CSF) and keratinocyte chemoattractant (KC) and the chemokines CCL2, and CCL5 have been reported to be secreted into luminal and abluminal chambers from primary mouse BECs in monoculture at baseline [86]. In tri-cultures that include primary mouse BECs, pericytes, and astrocytes, six additional cytokines and chemokines were found to be secreted: IL-6, IL-13, TNF-α, CCL11, CCL3, CCL4. All but CCL11 were predominantly secreted into the abluminal chamber. Secretion of these and additional cytokines were found to increase with LPS, with a tendency of LPS applied to the luminal side to increase secretion into the luminal chamber, and LPS applied to the abluminal side to increase secretions into the abluminal chamber, thus illustrating a polarization to secretion [86]. Such polarization could be important for relaying inflammatory signals to brain or blood compartments, or for establishing chemokine gradients that direct immune cell trafficking and migration [107, 108], which incorporates Axis 4. It has also been shown that the pro-inflammatory cytokines TNF-α, IL-1β, and IFN-γ can induce CCL2 expression in BECs and astrocytes [108]. TNF-α induction of BEC chemokines was shown to be mediated in part through GSK3β [109], and the same study also showed that inhibition of GSK3β reduced BBB disruption and leukocyte adhesion, highlighting the coordination of Axis 1 and Axis 4 with Axis 5. As described in Axis 2, secretions of cytokines such as IL-6 and GM-CSF in response to LPS can mediate biological functions such as HIV-1 transport and these responses can also be polarized [83]. Pericyte secretions of CCL2 can also enhance the effect of LPS on HIV-1 transport [84]. BECs also secrete other immune mediators such as prostaglandins and nitric oxide, both constitutively and in response to stimulation [1].

Extracellular vesicle shedding is another process that can be considered as an immune secretion. As an example, claudin 5-positive leukocytes emerge in the brain and blood in experimental autoimmune encephalomyelitis (EAE), which were found to occur from uptake of claudin-5 extracellular vesicles shed from brain endothelial cells. The functional significance of leukocyte acquisition of claudin 5 remains to be determined [110], but BEC shedding of TJP positive extracellular vesicles seems to be associated with other brain injury responses as well [111]. More complex functions of BBB secretions that integrate other immune axes have been described previously [1].

In summary, Axis 5 highlights that BECs have the ability to relay immune signals from blood-to-brain and perhaps also from brain-to-blood through their secretions. To what extent these secreted factors act by autocrine, juxtacrine, paracrine, and endocrine mechanisms remains an area of active study. Incorporation of different cell types of the NVU in co-culture with BECs may have important effects on their secreted immune factors.

## Neuroimmune studies in novel cellular/microfabricated platforms for studying the BBB

In the past decade, great advances have been made in the development of novel in vitro BBB models that more closely reflect the condition of the BBB in vivo. Perhaps the most beneficial was the development of an iPSC-derived model of BECs (iBECs) that develop high TEER values that can readily exceed 1000 Ω*cm^2^ and express functional BBB transporters. The first iteration of this model was published by Lippman et al. [4], and subsequent refinements to the iBEC culture protocol have resulted in improvements in TEER that can now exceed 5000 Ω*cm^2^ and low permeability to small tracers like sodium fluorescein around 2 × 10^−7^ cm/sec [112, 113]. Refinements have also included a shortened differentiation time [112], fully defined medium conditions [113], and procedures for co-culture of multiple other cell types of the NVU [114], including an isogenic model in which all cell types are derived from the same donor iPSCs [115]. iPSC-derived models of the BBB have given researchers the power to investigate disease mechanisms [116–118], the transport efficiency of specific drugs across the BBB [45, 119], and the impact of cell–cell interactions between BECs and other cells of the NVU such as astrocytes and pericytes [120, 121].

iBECs offer several advantages over conventional in vitro models of the BBB. First, iPSCs can be derived from human somatic cells such as fibroblasts, which are obtained by moderately invasive (e.g. skin punch biopsy) procedures while the donor is still alive. While fibroblasts are still the predominant cell type used for iPSC derivation, other cell types such as T-cells, epithelial cells, and keratinocytes can also generate iPSCs and obtaining them requires minimally invasive procedures [122]. Second, iPSCs can be expanded to generate a tremendous amount of starting material for iBEC differentiation and remain relatively consistent in their ability to differentiate into brain endothelial-like cells over many passages [4]. Third, genetic manipulations of iPSCs using gene editing approaches like CRISPR are available and can be used to introduce specific mutations into iBECs for studying gene functions or disease-specific mutations [123]. Fourth, iBECs recapitulate many of the phenotypes present in primary BECs, such as high TEER (> 1000 ohms*cm^2^ when differentiated in the presence of retinoic acid), low permeability, and proper expression, localization, and function of TJPs and some BBB transporters. However, some limitations of the model also exist such as the need for seeding density optimization to ensure proper differentiation [124], batch-to-batch differences in commercial reagents that can contribute to variable results, variability in barrier phenotypes of different iPSC lines [125], and a limited knowledge of developmental lineage effects [126]. There is also much knowledge to be gained about neuroimmune functions of iBECs, and the existing literature is discussed below.

A recent study by Mantle and Lee [127] investigated the effects of neuroinflammation in human iBECs with and without co-culture of human iPSC-derived astrocytes. In the monoculture model, it was shown that 24 h after the addition of TNF-α or TNF-α and IL-6 on the luminal and abluminal sides, TEER decreased when compared to control treatments. IL-6 treatment alone had no effect and TNF-α + IL-6 treatment had a comparable effect to TNF-α alone, suggesting that TNFα mediated the drop in TEER in this system. Interestingly, both TNF-α and IL-6 increased the permeability to sodium fluorescein and reduced the activity of P-gp. Therefore, both TNF-α and IL-6 had activities in monocultured iBECs, and IL-6 mediated changes in BBB leakiness that occurred in the absence of changes in TEER. The addition of astrocytes enhanced the TEER of the model as compared to the monoculture control. Astrocytes also mitigated the TEER-reducing effects of TNF-α and IL-6 co-treatment. In this study, cytokine profiles were evaluated from abluminal medium collected from iBECs in monoculture or co-culture with astrocytes following treatment with TNF-α + IL-6 or vehicle. iBECs produced detectable levels of CCL2 and IP-10 at baseline, and IFN-α and IL-8 became detectable in monocultured cells treated with TNF-α and IL-6. CCL2 expression also increased in the presence of TNF-α and IL-6. Co-culture with astrocytes in the presence of TNF-α and IL-6 treatment tended to increase the detectable numbers of pro-inflammatory mediators, which could be due either to astrocytes secreting cytokines or iBECs producing higher levels of cytokines in the co-culture system or both. Interestingly, despite increased production of many pro-inflammatory cytokines and chemokines with TNF-α and IL-6 treatment in the co-culture system, TEER reductions and increases in IgG permeability were inhibited by astrocyte co-culture. Therefore, the cytokines that were more potently induced in co-culture had minimal effects on BBB disruption and perhaps even promoted BBB integrity in this context.

A similar study by Linville et al. investigated the effects of inflammation on a 3D model of the BBB, where iBECs were seeded into a type I and IV collagen-coated hollow channel that is about 100 µm in diameter [128]. This model introduced a physiological shear stress to better mimic the physical environment of the BBB. To induce inflammatory conditions, iBECs were perfused luminally with TNF-α for 12 h and BBB permeability to inert tracers and aspects of leukocyte trafficking were assessed. TNF-α treatment upregulated the expression of ICAM-1 and VCAM-1, and the number of peripheral blood mononuclear cells (PMBCs) adhered to the iBECs was significantly increased. No apparent diapedesis occurred during the window of observation. TNF-α treatment alone did not alter the permeability of the iBBB to Lucifer yellow, Rhodamine 123, or 10 kDa dextran. These results are consistent with those described above for mouse BECs grown in a flow system [101], but differ from results reported from 2D-cultured iBECs results from Mantle and Lee [127]. Therefore, there may be important differences in iBEC responsiveness to cytokines depending on experimental conditions such as flow and polarity of application (e.g. luminal vs. abluminal). It may also be possible that batch effects of TNF-α, sizes of tracers used, and the differences between iPSCs lines used could be contributing to the apparent differences in immune responses among models.

One additional study that provides some technical insight on 3D modeling of the human BBB is that published by Brown et al. [129]. This study uses a sophisticated chip device where most components of the NVU are represented (i.e. primary human brain endothelial cells, astrocytes, pericytes, and iPSC-derived human neurons and astrocytes). Further, the luminal and abluminal compartments of this device can be sampled, and TEER measurements are possible in addition to assays of tracer permeability. TEER was rather low in this model (reported as 100 Ω), which may have been due to the use of commercially sourced primary human BECs. However, LPS treatment on the luminal side decreased TEER and increased permeability of the BECs, as did a cytokine cocktail. LPS induced cytokine changes in the vascular and brain chambers that included increases in GM-CSF, IL-17A, and TNF-α. One important technical point from this study was the realization that cytokines and LPS that were perfused into the system could strongly adsorb onto the cell-free device, and therefore reduced effective concentrations of cell treatments. This highlights an important control when novel materials are used to construct chip devices: some recombinant proteins are notoriously sticky and proper quality controls are needed to confirm the true treatment concentrations.

In summary, the studies using iBEC have currently investigated 4 of the 5 axes of BEC neuroimmune functions. The newer iBECs and other novel models such as the 3D culture systems have so far been shown to recapitulate some aspects of neuroimmune interactions known to occur in conventional in vitro BBB models and in vivo. A summary of the work with these newer models is:

- Axis 1: iBECs that form tight barriers in 2D exhibit disruption by pro-inflammatory cytokines, which is mitigated somewhat when in co-culture with astrocytes. iBECs in 3D were shown to be resistant to disruption with TNF-α application on the luminal side. Disruption can be detected following LPS treatment in primary human BECs in 3D, which are more leaky at baseline.
- Axis 2: Pro-inflammatory cytokine treatment of iBECs grown in 2D can contribute to reductions in P-gp function, and induced secretion of IFN-a, IL-8, and CCL2.
- Axis 3: Not yet described.
- Axis 4: 3D iBECs under flow conditions upregulate cell adhesion molecules that facilitate leukocyte adhesion, but not diapedesis following TNF-α treatment.
- Axis 5: 2D iBEC models were shown to secrete CCL2 and IP-10 at baseline.

## Conclusions

Together, these studies demonstrate that in vitro BBB models can be suitable platforms for studying the 5 axes of BEC neuroimmune functions. It is apparent that functional aspects of some neuroimmune axes in both conventional and newer in vitro BBB models may differ as a result of model construction (e.g. cell source or differentiation method of iBECs, 2D vs. 3D culture, co-culture with glia, cytokine sources, etc.). Recent advances such as improved tools for the accessibility and use of gene expression datasets have provided an added depth in the understanding of the BEC transcriptomic identity in vitro and in vivo [85, 126, 130–132]. When validated using functional assays, transcriptomics is a potentially powerful tool that could be used to make more comprehensive assessments of the BBB phenotype in vivo. Future work is needed to comprehensively evaluate the utility of iBECs and other advanced models of the BBB/NVU for studies of neuroimmune communication, as well as their limitations. Advances in this field using new cellular and molecular tools has great potential to facilitate our understanding regarding neuroimmunological functions of the BBB and NVU.

## Data Availability

Not applicable.

## Abbreviations

BBB: Blood–brain barrier
BEC: Brain endothelial cell
CCL: C–C motif chemokine ligand
CINC: Cytokine-induced neutrophil chemoattractant
DARC: Duffy antigen/receptor for chemokines
EGF: Epidermal growth factor
EV: Extracellular vesicles
FGF: Fibroblast growth factor
G-CSF: Granulocyte colony-stimulating factor
GM-CSF: Granulocyte-macrophage colony-stimulating factor
iBEC: Induced pluripotent stem cell-derived brain endothelial cell
ICAM: Intercellular adhesion molecule
IFN: Interferon
IL: Interleukin
iPSC: Induced pluripotent stem cell
JAM: Junctional adhesion model
KC: Keratinocyte chemoattractant
LFA: Lymphocyte function-associated antigen
LIF: Leukemia inhibitory factor
LPS: Lipopolysaccharide
NVU: Neurovascular unit
PBMC: Peripheral blood mononuclear cells
Pe: Permeability-surface area coefficient of the endothelial cell monolayer
P-gp: P-glycoprotein
TEER: Transendothelial electrical resistance
TJP: Tight junction protein
TLR4: Toll-like receptor 4
TNF-a: Tumor necrosis factor alpha
VCAM: Vascular cell adhesion molecule
